# PHYSICAL FUNCTION AMONG OLDER ADULTS AND CAREGIVERSARTHRITIS AMONG CAREGIVERS—BRFSS 2017, 2019

**DOI:** 10.1093/geroni/igac059.1193

**Published:** 2022-12-20

**Authors:** Eva Jackson, Michael Boring, Lisa McGuire, John Omura, Benjamin Olivari, Erica Odom, Janet Croft

**Affiliations:** Alzheimer's Association, Chicago, Illinois, United States; CDC, Atlanta, Georgia, United States; CDC, Atlanta, Georgia, United States; CDC, Atlanta, Georgia, United States; CDC, Atlanta, Georgia, United States; CDC, Atlanta, Georgia, United States; CDC, Atlanta, Georgia, United States

## Abstract

Unpaid caregivers are people who provide support to a family member or friend with a health condition or disability. While there are benefits to caregiving, it can negatively affect caregivers’ physical and mental health. Arthritis and caregiving affect 58.5 million and 53 million U.S. adults, respectively, and these numbers are expected to increase with increasing numbers of older adults. Little is known about the experience of caregivers with arthritis.Data from the 2017 and 2019 BRFSS were combined for all states that administered the optional Caregiving and the core Arthritis modules (17 states; n=91,224). Analyses accounted for the complex sampling design using SUDAAN (version 11.0; RTI International). Statistical significance was determined at α=0.05.Overall, 20.6% of respondents were caregivers, among whom 35.1% had arthritis compared with 24.5% of non-caregivers (p < 0.001). Arthritis among caregivers was higher in all 17 states compared to non-caregivers. Among adults with arthritis, caregivers were more likely to be younger, female, White, and more physically active than non-caregivers. Caregivers were also more likely to experience arthritis-related activity and work limitations than non-caregivers. Arthritis-related limitation outcomes varied at the individual state levels.Among caregivers, those with arthritis provided more care in weekly hours and duration (years) and were more likely to report difficulty walking or climbing stairs, dressing or bathing, and doing errands alone than caregivers without arthritis. Given the disparities between caregivers with and without arthritis, it is crucial for public health to collect data on the experience of caregivers with arthritis.

